# Inequalities in mortality associated with housing conditions in Belgium between 1991 and 2020

**DOI:** 10.1186/s12889-022-14819-w

**Published:** 2022-12-20

**Authors:** Martina Otavova, Christel Faes, Catherine Bouland, Eva De Clercq, Bram Vandeninden, Thierry Eggerickx, Jean-Paul Sanderson, Brecht Devleesschauwer, Bruno Masquelier

**Affiliations:** 1 Center for Demographic Research, UCLouvain, Louvain-La-Neuve, Belgium; 2 Data Science Institute, I-BioStat, Hasselt University, Hasselt, Belgium; 3 Department of Epidemiology and Public Health, Sciensano, Brussels, Belgium; 4 Research Centre On Environmental and Occupational Health, School of Public Health, Université Libre de Bruxelles, Brussels, Belgium; 5 Department of Risk and Health Impact Assessment, Sciensano, Brussels, Belgium; 6 Department of Translational Physiology, Infectiology and Public Health, Ghent University, Merelbeke, Belgium

**Keywords:** Housing inequality, Mortality, Health inequality, Belgium

## Abstract

**Background:**

Poor housing conditions have been associated with increased mortality. Our objective is to investigate the association between housing inequality and increased mortality in Belgium and to estimate the number of deaths that could be prevented if the population of the whole country faced the mortality rates experienced in areas that are least deprived in terms of housing.

**Methods:**

We used individual-level mortality data extracted from the National Register in Belgium and relative to deaths that occurred between Jan. 1, 1991, and Dec. 31, 2020. Spatial and time-specific housing deprivation indices (1991, 2001, and 2011) were created at the level of the smallest geographical unit in Belgium, with these units assigned into deciles from the most to the least deprived. We calculated mortality associated with housing inequality as the difference between observed and expected deaths by applying mortality rates of the least deprived decile to other deciles. We also used standard life table calculations to estimate the potential years of life lost due housing inequality.

**Results:**

Up to 18.5% (95% CI 17.7–19.3) of all deaths between 1991 and 2020 may be associated with housing inequality, corresponding to 584,875 deaths. Over time, life expectancy at birth increased for the most and least deprived deciles by about 3.5 years. The gap in life expectancy between the two deciles remained high, on average 4.6 years. Life expectancy in Belgium would increase by approximately 3 years if all deciles had the mortality rates of the least deprived decile.

**Conclusions:**

Thousands of deaths in Belgium could be avoided if all Belgian neighborhoods had the mortality rates of the least deprived areas in terms of housing. Hotspots of housing inequalities need to be located and targeted with tailored public actions.

**Supplementary Information:**

The online version contains supplementary material available at 10.1186/s12889-022-14819-w.

## Background

According to the United Nations, the right to housing is a basic human right, not just to a ‘basic shelter’ but to ‘adequate housing’ in terms of legal security of tenure, availability of services, materials, facilities and infrastructure, affordability, habitability, accessibility, location, and cultural adequacy [1]. Although improvements in housing have been central to many social policy interventions and are fundamental to tackling elements of poverty, over a billion people worldwide remain inadequately housed.

Housing affects health in numerous minor ways and can be considered an intermediate social determinant of health because it links poverty and well-being through various mechanisms [2, 3]. Epidemiological research suggests that poor housing can have a direct and indirect effect on health and mortality [4]. The direct effects may result from the material conditions of housing. For instance, depending on the climatic context, housing can, more often than not, be too cold or too hot for healthy living due to lack of ventilation, central heating, or air conditioning. Cold and humid environments caused by inadequate ventilation create conditions for the growth of mold and fungi that are associated with the incidence of diarrhea, headaches, fever, and respiratory diseases [5–7]. Furthermore, exposure to extreme cold and the absence of central heating is linked to a higher risk of dying in winter, whereas lack of air conditioning during extreme heat waves results in increased mortality during the summer months [8, 9]. In addition to the material dimension, housing also has symbolic significance, referring to the fact that a house represents a place of refuge, is an essential part of one’s identity, and plays a role in social interactions. Poor housing and a poor neighborhood environment may lead to pessimism and passivity, chronic stress, and a state of personal dissatisfaction – conditions that negatively affect physical and mental health [4, 10–12].

The neighborhood in which housing is located and its natural and built environment, as well as the social and cultural norms present there, represent other indirect ways in which housing affects health [4]. For instance, in neighborhoods with high smoking rates, smoking might be considered the norm and deviation from such behavior a weakness [13].

Despite the wide range of possible negative impacts of inadequate housing on health, only a handful of studies have considered the relationship between housing conditions and variation in life expectancy. Housing tenure, one of the most commonly used indicators of housing and socio-economic status, has been found by several researchers to be associated with health outcomes. For instance, Filakti et al. reported that in England and Wales premature mortality was 35% lower for male home owners than for males living in social housing in the 1980s [14], and, more recently, a study in Portugal reported that living in social housing is associated with increased mortality [15]. Strong associations between housing tenure and increased mortality or poor health were also reported by studies from Scotland [16] and the US [17], while findings from Australia suggest that housing affordability stress has adverse impacts on wellbeing, especially mental health [18]. Findings from existing studies on the association between inadequate housing conditions in Belgium and adverse health outcomes are concurrent with findings reported worldwide [19–21]. For instance, a study by Damiens (2020) showed that mortality among tenants was about 25% higher than among home owners, and a similar excess mortality was observed among those living in low-quality housing, defined by lack of elements such as a bathroom, central heating, or a kitchen, even after controlling for educational attainment, professional status, and income [20].

Although the Belgian Constitution includes the right to decent housing, and Wallonia, Flanders, and the Brussels-Capital Region share a common social policy, access to housing remains a major social differentiator [22–24]. The differences in housing quality among the three regions are large, with housing quality being highest in the Flemish region and lowest in the Brussels-Capital Region, where the number of homeless and inadequately housed people has more than doubled since 2008 [19–21, 25]. A strikingly similar pattern in mortality was reported by Eggerickx et al. (2019), showing that, between 1991 and 2016, the highest levels of mortality were in the inner city of the Brussels-Capital Region and in several Walloon cities, compared to the Flanders [26].

The objective of this paper is to assess whether housing inequality is associated with increased mortality and to estimate the number of deaths that could be prevented if the entire population of Belgium faced the mortality rates of the least deprived areas in terms of housing. We use housing conditions as concrete manifestations of socio-economic deprivation and create composite, spatial, and time-specific housing deprivation indices for measuring deprivation levels at the smallest administrative unit in Belgium, the statistical sector. Then, we perform an examination of the association between housing inequality and mortality, using multiple measures of health inequality.

## Methods

### Housing Deprivation Indices (HDI)

We used data on housing characteristics from the 1991, 2001, and 2011 Belgian population censuses to develop area-level housing deprivation indices at the level of statistical sector for these three census years. Each index was built on a group of indicators encompassing multiple deprivation aspects. The selection of indicators was based on our literature review and on the premise that the indicators must be the best possible measure of housing deprivation in Belgium for the given time period. The indicators retained vary across the selected years due to their relevance at the time, accessibility, and availability, and they always represent only the proportion of population who is deprived, not affluent (Table 1).

**Table 1:** Overview of indicators used for the 1991, 2001, and 2011 housing deprivation indices and prevalence in the population of Belgium for the given year

		**1991**			**2001**			**2011**	
	**% in Belgium**	**Missing data (%)**	**Total population**	**% in Belgium**	**Missing data (%)**	**Total population**	**% in Belgium**	**Missing data (%)**	**Total** **population**
**Tenants**	29.6	3.8	9,469,913	26.5	4	9,726,611	30.5	1.03	10,985,250
**Property size less than 35m**^**2**^	2.8	3.6	9,486,459	6.1	7.8	9,348,105			
**No central heating**	36.5	4.2	9,427,568	25.4	4.7	9,655,456	12.8	5.8	10,361,725
**No toilet**	6.5	3.6	9,487,524	2.9	5.4	9,586,814			
**No bathroom**	8.5	3.8	9,467,117	2.9	4.7	9,655,346	1.1	4.2	10,538,313
**No landline**	10.7	5.2	9,332,268						
**No insulation**				25.1	5.7	9,554,520			
**No internet**				65.9	9.4	9,175,109			
**No kitchen**	4.1	2.0	9,644,084	14.9	7.9	9,325,967			
**Less than 0.5 rooms per person**							5.1	2.7	10,704,763

For each indicator, the proportion of the population in a statistical sector that is deprived was calculated, for instance the proportion of individuals without central heating or internet connection. Maximum likelihood factor analysis (FA) was used to combine these indicators at the level of statistical sector. Then, statistical sectors were ranked based on the resulting FA scores and assigned to deciles such that the most deprived statistical sectors fell into the first decile. This methodology was applied independently for the 1991, 2001, and 2011 indices. More details are provided in the Supplementary Information.

### Geographical unit

The HDIs are presented at the level of statistical sector, which is the smallest administrative unit of Belgium, resulting from the subdivision of municipalities [27]. By definition, a statistical sector may never extend over two municipalities, and each geographical point in Belgium belongs to one and only one statistical sector [28]. These sectors are heterogeneous in population size, with a median population of 300 persons.

In our study, only statistical sectors with more than 10 inhabitants were included to ensure obtaining stable estimates, to limit potential bias, and to safeguard data privacy. We excluded 1,464 (7.5%), 1,486 (7.5%), and 1,018 (5.1%) statistical sectors for 1991, 2001 and 2011 respectively. The final number of statistical sectors included in the analysis was therefore 17,909 for the period 1991–2000, 18,295 for the period 2001–2010, and 18,764 for the period 2011–2020.

### Data

We used a pseudonymized dataset, built upon DEMOBEL, the demographic database produced by the Belgian Statistical Office [26, 29]. Our database covers the observation period 1991–2020 and includes several administrative data sources: the National Register, the 1991, 2001, and 2011 Belgian population censuses, and death certificates from the Civil Registry. Individuals in these data sources are linked by a multi-digit code specific to the research project. The pseudonymization prevents linkages of the data with other administrative databases or databases located in other research centers.

### Missing data

The amount of missing information varied across indicators and censuses – 4%–12% for 1991, 6%–17% for 2001, and 2%–12% for 2011. Whenever possible, we identified individuals within the same household and imputed missing information based on other family members. Additionally, we imputed values based on the same housing profile – individuals in the same statistical sector had to share at least 75% of housing characteristics listed in Table 1. These two approaches resulted in a reduction of missing values to 2%–5% for 1991, 4%–9% for 2001, and 1%–6% for 2011 (Table S2 in Supplementary Information).

### Mortality data

We used pseudonymized individual-level all-cause mortality data extracted from the National Register in Belgium relative to deaths that occurred between Jan. 1, 1991, and Dec. 31, 2020. Data included age, sex, and a unique personal identification number for each deceased individual. Multi-digit identification numbers were used for record linkage within the database to derive the last registered statistical sector of the deceased. Mid-year population estimates by statistical sector, sex, and single-year of age from 1991 to 2020 were obtained from the National Register data, which includes all legal residents of Belgium and excludes irregular migrants and asylum seekers.

### Inequality indices

First, we stratified the mortality and population data according to 5-year age group (with the lowest age group 0–4 and the open-ended age interval 95 +), sex, housing deprivation deciles, and 10-year periods (1991–2000, 2001–2010, 2011–2020), yielding 1,260 strata. Housing deprivation indices were used as follows: the HDI 1991 was applied to the period 1991–2000, the HDI 2001 to 2001–2010, and the HDI 2011 to 2011–2020. We then computed sex- and age-standardized mortality rates (ASMR) per 100,000 person-years and at 95% confidence intervals (CI) for each stratum using the sex and age structure of the Belgian population in 2019. To show the changes over time, we calculated the absolute change in ASMR between the first (1991–2000) and last period (2011–2020). We plotted the adjusted Lorenz curve and computed Gini coefficients for each period to observe inequalities in ASMRs across the housing deprivation deciles. The Lorenz curve is a cumulative frequency curve [30] that, in our case, compares the distribution of sex- and age-standardized deaths across deciles, with a uniform distribution representing equality. The adjusted Lorenz curve was plotted by using the cumulative proportion of sex- and age-specific deaths and cumulative proportion of population by housing deprivation deciles. We used the ASMRs to compute the sex- and age-specific number of deaths in each decile and consequently computed the cumulative percent of deaths. An example of our calculations is provided in Table S4 in Supplementary Information. Gini coefficients were computed using the Gini function from the DescTools package in R [31].

Secondly, within each 5-year age group, sex, and 10-year-period, the age-specific mortality rate of the least deprived decile was used as a reference group and applied to the other deciles to produce a number of expected deaths. We calculated the mortality associated with housing deprivation as the difference between the observed and expected deaths, expressed as a population attributable fraction (PAF) and a number of excess deaths. The 95% CIs were estimated using a Monte Carlo simulation, in which 10,000 deaths in each stratum were sampled from a Poisson distribution.

Thirdly, we constructed period life tables for each sex, housing deprivation decile, and 10-year period [32]. The Arriaga method was used to decompose the differences in life expectancy between the most and least deprived deciles in men and women for all three decades separately. Finally, potential years of life lost (PYLLs) to inequality were calculated as the difference between the years lost due to death before age 75 in each cohort and the corresponding least deprived cohort.

All analyses were done in R version 4.1.1 [33].

## Results

**Fig. 1:**
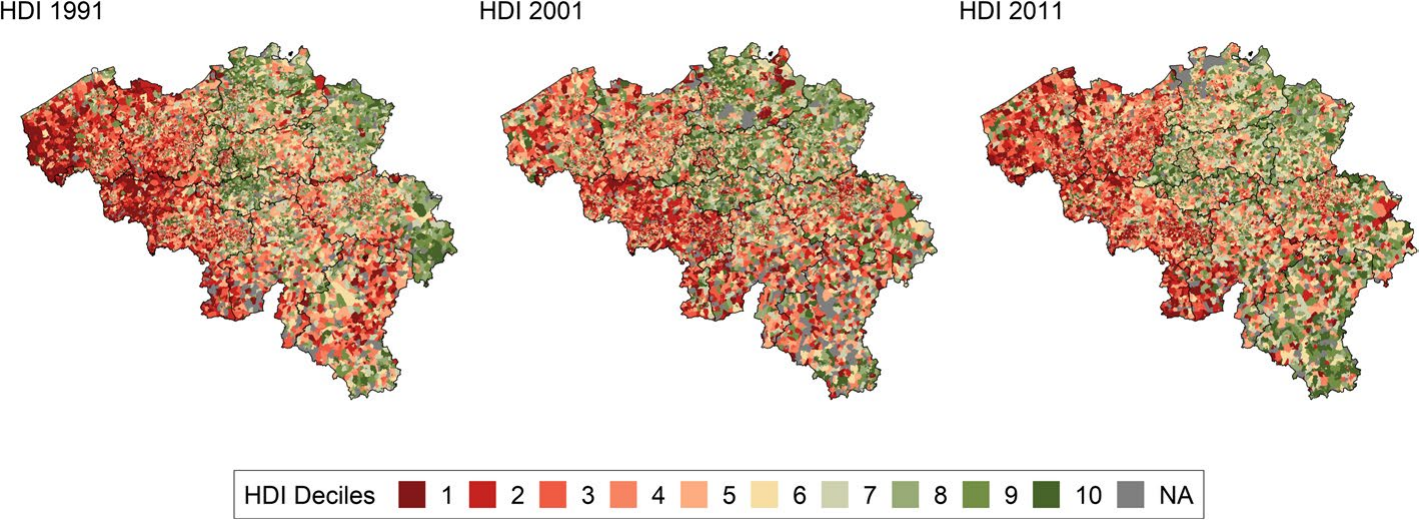
Distribution of housing deprivation deciles across Belgian statistical sectors in 1991, 2001, and 2011. The most deprived statistical sectors fall under the first deprivation decile (dark red)

Our study covered 310 million person-years in people aged 0–95 + , with 3,161,490 deaths that occurred in Belgium between 1991 and 2020. Deprivation was defined by deciles of housing deprivation indices for 1991, 2001, and 2011 at the level of statistical sector (Fig. 1). During the study period, ASMRs decreased for men and women in all deprivation deciles. Reduction in absolute ASMR was greatest for males in the most deprived decile (− 406 per 100,000 person-years) and females in the least deprived deciles (− 293 per 100,000 person-years), whereas relative reductions were greatest for males (− 31%) and females (− 25%) in the least deprived deciles (Table 2). Comparing the period 2011–2020 with 1991–2000, the gap between the most and least deprived deciles narrowed for men, but widened for women.

**Table 2:** Trends in mortality inequalities according to housing deprivation deciles

	**1991–2000**^**1**^	**2001–2010**	**2011–2020**	**Change (%)**^**4**^
Age-standardized mortality rates per 100,000 person-years and 95% CI^5^				
Male				
Most deprived^2^	1,649 [1634–1664]	1,495 [1484–1506]	1,243 [1231–1254]	− 24.62
Least deprived^3^	1,193 [1181–1207]	923 [911–935]	826 [815–837]	− 30.76
Difference	456	572	417	− 8.55
Female				
Most deprived	1,402 [1389–1415]	1,271 [1262–1281]	1,140 [1130–1150]	− 18.69
Least deprived	1,156 [1144–1170]	1,027 [1013–1041]	863 [851–875]	− 25.34
Difference	246	244	277	12.60
Gini coefficient				
Male	0.05 [0.03–0.08]	0.08 [0.06–0.12]	0.07 [0.05–0.10]	
Female	0.03 [0.02–0.05]	0.04 [0.03–0.05]	0.04 [0.03–0.07]	
Mortality associated with housing inequality (%)				
Male	21.0 [20.12–21.85]	26.5 [25.13–27.99]	19.4 [18.33–20.47]	-7.6
Female	12.6 [11.61–13.51]	11.27 [9.48–13.02]	15.5 [14.47–16.75]	23.2
Mean number of Potential Years of Life Lost associated with housing inequality^6^				
Male	1.50	1.58	0.92	− 38.7
Female	0.61	0.69	0.46	− 24.6

Mortality is adjusted to the Belgian population structure in 2019

^1^ Housing deprivation index 1991 applied for the period 1991–2000, index 2001 for 2001–2010, and index 2011 for 2011–2020

^2^ Most deprived refers to the most deprived decile of the housing deprivation indices for 1991, 2001, 2011

^3^ Least deprived refers to the least deprived decile of the housing deprivation indices for 1991, 2001, 2011

^4^ Change in mortality between 1991–2000 and 2011–2020

^5^ ASMRs calculated for the whole population of Belgium with an open-ended 95 + group

^6^ PYLLs associated with housing inequality were calculated for the population younger than 75 years

The adjusted Lorenz curves show that in 1991–2000, 9.9% and 9.1% of male and female deaths occurred in the most deprived decile, which only represents about 8.9% of the male and 8.5% of the female population. In the period 2001–2010, the first and most deprived deciles contained 13% and 12.5% of the male and female population, while accounting for 15.9% of male deaths and 13.9% female deaths. Between 2011–2020, the first decile contained similarly 7.7% of the male and female population and accounted for 9.3% and 8.6% of male and female deaths.

The Gini coefficient, measuring the degree of ‘bend’ or inequality in the Lorenz curve, was slightly fluctuating for men (without statistical significance) but stayed almost constant for women over the periods under observation. The Gini coefficients are provided in Table 2 and the adjusted Lorenz curves are shown in Figure S5 (in Supplementary Information).

The proportion of mortality associated with housing inequality varied by time period, sex, and age. Across all years under study, the PAF was higher in men than in women, first increasing sharply from 21.0% (95% CI, 20.1–21.9) to 26.5% (95% CI, 25.1–28.0) in the periods 1991–2000 and 2001–2011, then decreasing to 19.4% (95% CI, 18.3–20.5) in the period 2011–2020. In women, mortality associated with inequality has been increasing over time, from 12.6% (95% CI, 11.6–13.5) to 15.5% (95% CI, 14.5–16.8) between 1991–2000 and 2011–2020. In the most deprived deciles, the proportion of deaths associated with housing deprivation peaked in middle-aged men and women and accounted for more than half of all deaths occurring in the middle-aged population (Figs S1, S2, and S3 in Supplementary Information).

**Fig. 2:**
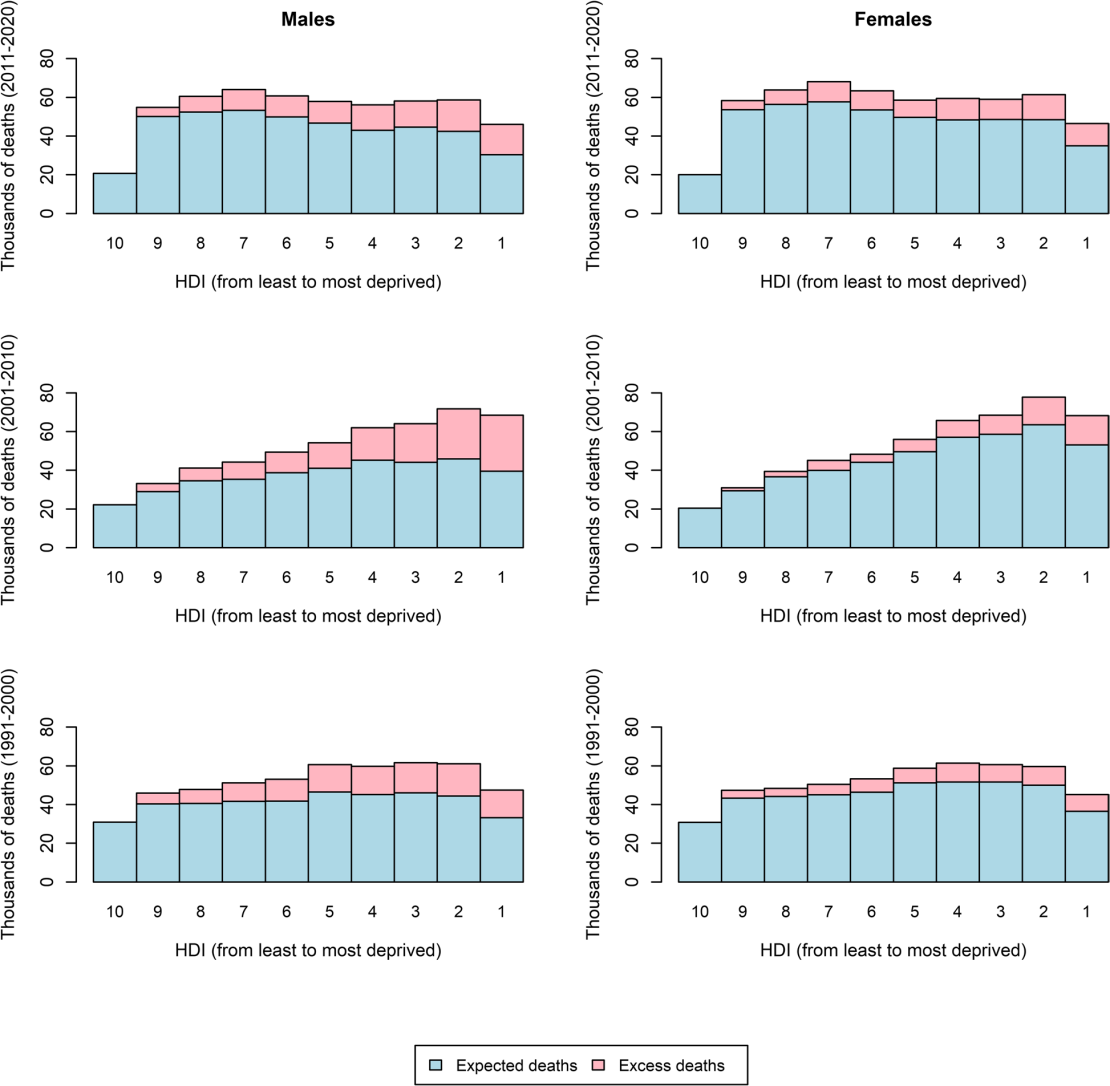
Mortality associated with housing inequality in Belgium by housing deprivation deciles

If the whole population of Belgium presented the same risk of mortality as the least deprived groups measured by the HDI in the three reference years 1991, 2001, and 2011, 568,605 fewer deaths would have occurred between 1991 and 2020. This represents about 18% of all deaths that could be associated with housing deprivation (Fig. 2).

The results of our life table calculation suggested that on average, men and women in the least deprived deciles live longer than in the most deprived deciles in all observed periods. For the period 1991–2000, men and women in the least deprived deciles were expected to live 78.1 and 82.8 years, respectively, compared to 72.7 and 79.9 years in the most deprived deciles (Table 3). The gap in life expectancy between the most and least deprived deciles was 5.4 and 2.9 years for men and women, respectively. In the subsequent periods of 2001–2010 and 2011–2020, the gaps between the most and least deprived groups for men and women increased to 7.1 and 4.0 years, and consequently decreased to 5 and 3.3 years, respectively.

**Table 3:** Life expectancies at birth in Belgium by the most and the least housing deprivation deciles for 1991–2000, 2001–2010, and 2011–2020

	**1991–2000**^**1**^	**2001–2010**	**2011–2020**	**Change in time** **(in years)**^**4**^
Male				
Most deprived^2^	72.72	73.90	76.98	4.26
Least deprived^3^	78.07	80.96	81.96	3.89
Difference	5.35	7.06	4.98	
Female				
Most deprived	79.88	80.68	82.53	2.65
Least deprived	82.78	84.65	85.84	3.06
Difference	2.90	3.97	3.31	

Mortality is adjusted to the Belgian population structure in 2019

^1^ Housing deprivation index 1991 applied for the period 1991–2000, index 2001 for 2001–2010, and index 2011 for 2011– 2020

^2^ Most deprived refers to the most deprived decile of the housing deprivation indices for 1991, 2001, and 2011

^3^ Least deprived refers to the least deprived decile of the housing deprivation indices for 1991, 2001, and 2011

^4^ Change in life expectancy at birth between 1991–2000 and 2011–2020

Over the three decades, life expectancy at birth increased for all deciles, but not at the same rate. Using the Arriaga method, we compared how differences in age-specific mortality contributed to the life expectancy gap between the most and least deprived deciles in each period under study. The increase in life expectancy is observed in all age groups, but it is driven mostly by declines in mortality in the age group 60–69 in men and 70–79 in women, in all three decades. These age groups contribute about 25% to the difference in life expectancy between the most and least deprived deciles in 1991–2000, 2001–2010, and 2011–2020 (Table S3 in Supplementary Information).

**Fig. 3:**
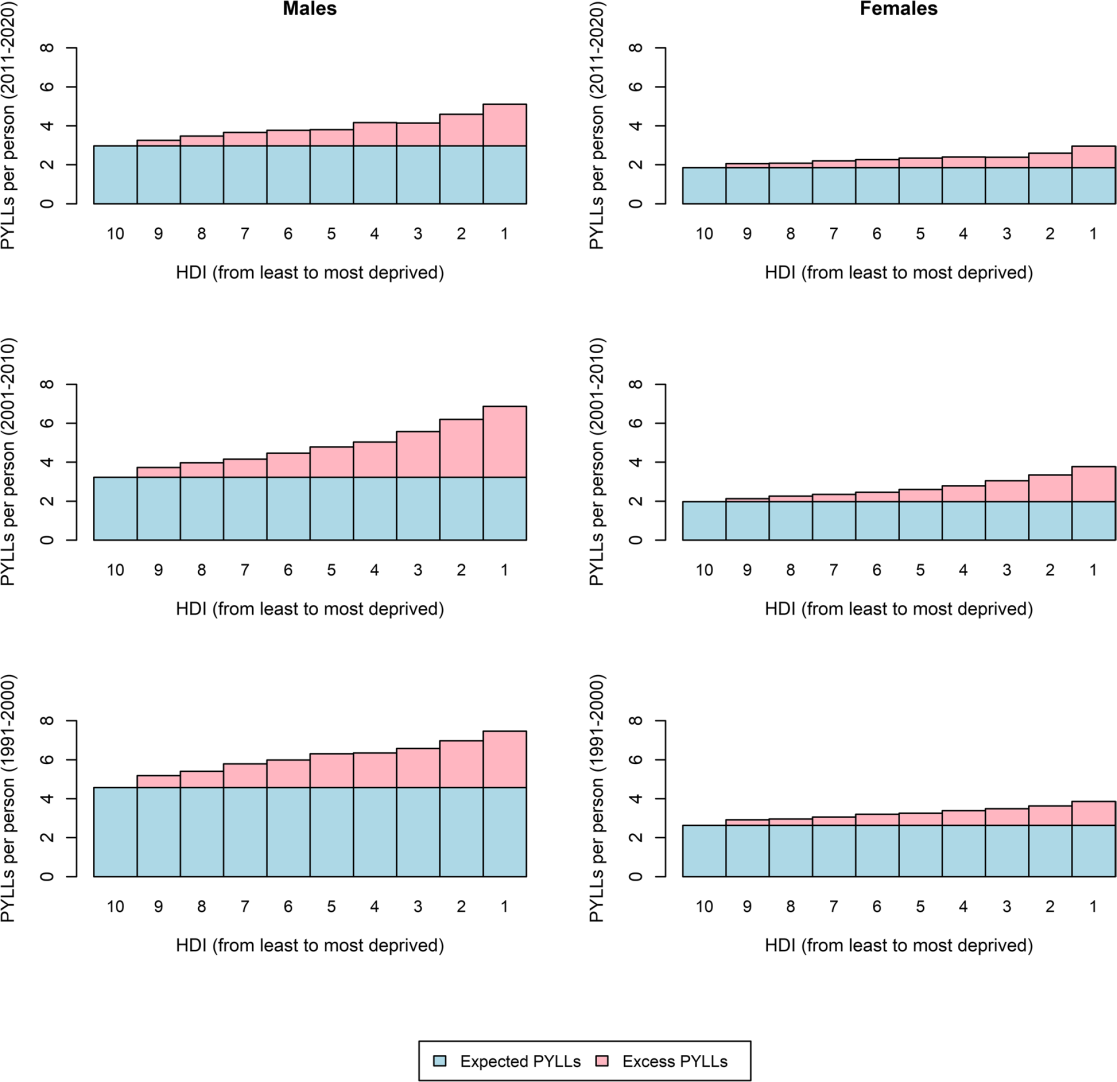
Potential years of life lost to housing inequality in Belgium by housing deprivation deciles

The results of our life table calculation also revealed that premature mortality associated with housing deprivation caused a mean of 4.7, 3.74, and 3.1 PYLLs per person in 1991–2000, 2001–2010, and 2011–2020 respectively. Sex-stratified results showed that the mean number of PYLLs has always been greater for men than women across all deciles, with a declining trend for both sexes (Fig. 3). The mean PYLL for men and women, respectively, was 6.1 and 3.2 for the period 1991–2000, 4.8 and 2.7 for 2001–2010, and 3.9 and 2.3 years for 2011–2020. If everyone had the mortality risk of the least deprived groups measured by the HDI for 1991, 2001, and 2011, the PYLLs would decrease to 3.6, 2.6, and 2.4 years per person. The average number of PYLLs associated with housing deprivation is shown for each sex in Table 2.

Our results also suggested that 23%, 30%, and 22% of PYLLs were associated with inequality for the years 1991, 2001, and 2011. In both sexes, this proportion peaked in the period 2001–2010, equally with 68% in men and women.

## Discussion

To our knowledge, this is the first Belgian study investigating housing-driven inequalities in mortality using composite, spatial, and time-specific housing deprivation indices. The HDIs were created using data from the 1991, 2001, and 2011 population censuses, and the units of analysis were the smallest administrative units in Belgium, statistical sectors.

We conducted a cross-sectional study of 3,161,490 deaths that occurred in Belgium between 1991 and 2020, and we showed that one in five deaths could be associated with inequalities in housing measured by the HDIs. During the study period, the ASMRs declined in both sexes across all deprivation deciles, but inequality in mortality persisted. The differences in the ASMRs between the most and least deprived deciles were greatest for men in the study period of 2001–2010 and women in 2011–2020. Comparing the periods 1991–2000 and 2011–2020, we observed that the gap in the ASMRs between the most and least deprived deciles of the HDIs and the mortality associated with housing inequality decreased in men but increased in women, whereas the mean number of PYLLs associated with housing inequality decreased in both sexes. Life expectancy at birth increased in men and women across all housing deprivation deciles, mostly driven by declines in mortality at higher ages. The disparities in life expectancy at birth between the most and least deprived deciles decreased in men and increased in women.

In the period 2001–2010, we observed opposite trends in mortality associated with housing inequality and in the PYLLs. In men, the mortality associated with housing inequality and the PYLLs increased, but in women, both decreased. In addition, the gap between the most and least deprived housing deprivation deciles for the ASMRs increased in men but decreased in women, and for the life expectancy at birth increased for both sexes.

Findings of our study are consistent with results of prior studies that investigated variations in life expectancy by housing quality and housing tenure. Eggerickx et al. (2018) showed an increase in social and spatial inequalities in Belgium between 1991 and 2016, pointing out that inequalities are greatest among individuals aged 25 to 50 [19], in line with our results depicting that mortality associated with housing inequality peaks between the ages of 40 and 49. The study by Damiens (2020) on the impact of housing conditions on mortality in Belgium concluded that low-quality housing may result in an increase in mortality rates by 25% after controlling for educational attainment, professional status, and income, whereas in our study, the difference between the most and least deprived groups ranged between 28%–40% for men and 18%–25% for women [20].

Although we are not able to explain why the time trends in inequality differ between men and women, our results might help to better understand the association between housing deprivation and health and guide housing policies. In Belgium, one weakness of public housing programs is the fact that housing policies are mostly centered on access to homeownership, while the stock of social housing remains insufficient. For instance, in Wallonia in 2007, 3.4% of households were awaiting social housing, and 75% of these were considered extremely poor [34]. A similar situation was observed in Flanders and the Brussels-Capital Region [35]. Moreover, the private rental sector is often characterized by poor housing conditions, as it receives the least public support [25]. Knowing that residing in inadequate housing is associated with adverse health outcomes, housing should be ranked a priority health determinant in regional and national policies and surveillance systems. Achieving improvement might require a coordinated cross-departmental approach involving different spheres of policy targeting social determinants of housing.

### Strengths and limitations

The key strength of our study is our development and use of spatial and time-specific indices of housing deprivation. The method we used to build our HDIs is easily replicable and can be updated in the future with the latest data. Indices as well as our population-level estimates of housing inequality in mortality were computed at the level of statistical sector, but they can be aggregated to higher-area levels, such as municipalities. Our decision to build the HDIs at the aggregate level was supported on several points. We were interested in capturing area deprivation effects as conceptualized in three different meanings: as compositional, as collective, and as environmental [36]. A compositional meaning of area deprivation refers to poor housing conditions that are ultimately experienced by individuals and further translated into proportions of deprived people living in an area. A collective meaning and an environmental meaning refer to additional deprivation in an area that is beyond that attributable to the concentration of deprived people; these can be seen as synonymous with neighborhood effects. Neighborhood effects have been increasingly researched, specifically in relation to health or housing, and studies have shown that neighborhoods affect their inhabitants, even after accounting for individual risk factors [4, 37–40]. Creating the HDIs at the level of statistical sector, we were able to include all three dimensions, but it became difficult to isolate the individual effect and the neighborhood effect, as the housing characteristics are constructed on the aggregation of individual variables and not on environmental variables. Finally, as the sectors’ characteristics are relatively stable over time, our aggregate approach allows for easier identification of areas in need of interventions that would benefit from area-based policies on housing and urban planning.

In choosing a suitable geographical area, we preferred the smallest administrative unit in Belgium, the statistical sector. This choice was also justified by the fact that statistical sectors are official administrative units; their boundaries change little over time; they are large enough to provide statistically robust estimates of housing or health indicators; and better homogeneity within their populations can be assumed, reducing the risk of ecological fallacy [41]. In addition, the use of aggregate data was more practical as it enabled us to better accommodate the privacy, security, and confidentiality issues associated with using administrative and survey data.

Another real advantage of our study is the use of data from the whole population of Belgium to obtain population-level estimates of inequality measures. Using data from the whole population allowed us to avoid selection bias. All-cause mortality data from death registrations are exhaustive and highly reliable in Belgium [42]. Our data can be broken down by geographical area to pinpoint the areas with the greatest health inequalities to ensure their sufficient allocation of public health resources.

This study has limitations that need to be considered when interpreting the results. While the statistical sector is the smallest geographical unit available in Belgium, we should be mindful of problems such as any remaining risk of ecological fallacy [43]. Inferences about individuals cannot be drawn from this analysis, as it looks at the effect of group-level characteristics. It would be false to assume that all individuals living in highly deprived areas must themselves be highly deprived, and conversely, that individuals living in relatively less deprived areas must themselves be less deprived.

The HDIs are built on housing characteristics that were recorded on the respective dates of the 1991, 2001, and 2011 population censuses, but they are applied to the 10-year periods that follow each of these. As Spearman’s rank correlations between the overall HDI scores were about 0.7 and correlations between the individual indicators mostly around 0.8 (Table S5 and Table S6 in Supplementary Information), we assumed that the HDIs are fairly stable over time. However, we cannot eliminate a change in the level of housing deprivation across statistical sectors during a 10-year period.

Another limitation of our study relates to the fact that information on housing conditions was collected differently in the three censuses. In 1991 and 2001, data were collected through an individual questionnaire, while in 2011 administrative databases were used to update the previously existing information. This difference in data collection can result in comparability issues. Another concern is the absence of information concerning individuals living in communal living facilities, such as nursing homes. The proportion of missing information on housing for those individuals was equal to 75%, 95%, and 89% in the 1991, 2001, and 2011 censuses respectively. For this reason, we have excluded them from our analysis. The amount of missing information on individual housing indicators differed highly across the three censuses. Linking the census data with the national register, we were able to impute missing information based on other family members living in the same household. In addition, we imputed values based on similar profiles – matching individual profiles within a given statistical sector. Although we were able to partially impute some information, missing values still ranged between 2 and 6% in 1991 and 2011, and between 4 and 10% in 2001. Ignoring the missing cases and including only legal residents in our analysis likely led to a slight underestimation of housing inequalities across Belgium. Non-response was shown to be selective in regards of vulnerable individuals of a lower socio-economic status or individuals in poor health [44]. Illegal residents of Belgium often reside in dwellings of modest or poor quality [45].

As we use housing conditions as a concrete manifestation of socio-economic status, a question of correlation between housing quality and other factors might arise. Previous studies showed that inequalities in housing are closely related to differences in income or education, and it is possible that some of the inequalities observed here refer to these dimensions. We are currently working on developing a Belgian index of multiple deprivation (BIMD) that integrates the issues of housing, income, education, employment, and crime. Tools such as the BIMD will enable us to isolate the specific contribution of housing conditions – a task currently beyond the scope of this study. It is also very likely that the impact of housing deprivation on health is mediated by lifestyle factors such as tobacco smoking or alcohol consumption. People living in deprived circumstances generally have higher tobacco and alcohol consumption [46], and thus, some of these deaths could also be attributed to housing deprivation [47]. Moreover, a debate exists about the extent to which poor health precedes or results from inadequate housing, and evidence supporting both causal directions exist; however this is not a key issue in our study because either pathway confirms the close relationship between inadequate housing and health [4].

We aimed to measure the extent of health inequality associated with housing conditions rather than capture a causal effect of housing deprivation on health. The design of our cross-sectional study does not allow us to assess the causal relationship between housing deprivation and mortality rates and is not suitable for showing that any of the indicators of the HDIs have caused death or that their improvement would result in a decrease in mortality. Instead, we showed a scenario in which everyone in Belgium the same mortality as the least deprived decile of the HDIs, without suggesting how this scenario could be achieved. By its very nature, the study is designed to show the scale of health inequalities associated with inadequate housing rather than the cause of the health inequalities.

## Conclusions

Our findings provide a better understanding of the extent to which housing inequalities are associated with mortality and suggest that important socio-spatial inequalities still exist in Belgium. Using composite housing deprivation indices and multiple measures of health inequality, we showed that every year thousands of deaths in Belgium could be avoided if Belgium had the mortality rate of the least deprived decile. Finally, our findings can help the Belgian government and local politicians to locate hotspots of poor housing and their associated health inequalities for better targeted public action.

## Supplementary information

**Additional file 1: Table S1 **Indicator weights generated by factor analysis for the individual years. **Table S2 **Missing values across indicators prior and after imputing based on similar profile. **Figure S1** Percentage of mortality attributable to housing inequality by the HDI 1991, sex and age group in a period of 1991-2000. **Figure S2 **Percentage of mortality attributable to housing inequality by the HDI 2001, sex and age group in a period of 2001-2010. **Figure S3 **Percentage of mortality attributable to housing inequality by the HDI 2011, sex and age group in a period of 2011-2020. **Table S3 **Results of the Arriaga method. Absolute contribution represents the sum of the direct and indirect effects of an age group between the most and the least deprived deciles in each period.** Table S4 **Example of our calculations used for plotting the adjusted Lorenz curve. **Figure S4 **Adjusted Lorenz curve for age-adjusted male mortality in the period between 1991-2000. **Table S5 **Spearman’s correlation coefficients between the indicators of the HDI 1991 and HDI 2001. Table S6 Spearman’s correlation coefficients between the indicators of the HDI 2001 and HDI 2011.

## Data Availability

The data that support the findings of this study are available at the Belgian statistical office, Statbel, but restrictions apply to the availability of these data, which are used under license for the current study, and so are not publicly available. Data are however available from the corresponding author upon reasonable request and with the permission of Statbel.
