# Supplementary material for: Inequalities in mortality associated with housing conditions in Belgium between 1991 and 2020
Source: BMC Public Health. 2022 Dec 20;22:2397. doi: 10.1186/s12889-022-14819-w (PMC9769013; doi:10.1186/s12889-022-14819-w)
Supplement: Supplementary file 1 — Additional file 1: Table S1 Indicator weights generated by factor analysis for the individual years. Table S2 Missing values across indicators prior and after imputing based on similar profile. Figure S1 Percentage of mortality attributable to housing inequality by the HDI 1991, sex and age group in a period of 1991-2000. Figure S2 Percentage of mortality attributable to housing inequality by the HDI 2001, sex and age group in a period of 2001-2010. Figure S3 Percentage of mortality attributable to housing inequality by the HDI 2011, sex and age group in a period of 2011-2020. Table S3 Results of the Arriaga method. Absolute contribution represents the sum of the direct and indirect effects of an age group between the most and the least deprived deciles in each period. Table S4 Example of our calculations used for plotting the adjusted Lorenz curve. Figure S4 Adjusted Lorenz curve for age-adjusted male mortality in the period between 1991-2000. Table S5 Spearman’s correlation coefficients between the indicators of the HDI 1991 and HDI 2001. Table S6 Spearman’s correlation coefficients between the indicators of the HDI 2001 and HDI 2011. [file 12889_2022_14819_MOESM1_ESM.docx]

# Supplementary materials

Supplement to: Otavova M, Faes C, Bouland C, De Clercq E, Vandeninden B, Eggerickx T, Sanderson P, Devleesschauwer B, Masquelier B., Inequalities in mortality associated with housing conditions in Belgium between 1991 and 2020

1. Housing deprivation indices 1991, 2001, and 2011
   1. Indicators
   2. Factor analysis
   3. Missing data
2. Mortality attributable to housing inequality
3. The Arriaga decomposition
4. Lorenz curve and GINI coefficient
5. Spearman’s Rank correlation

# Housing deprivation indices 1991, 2001, and 2011

## Indicators

The Housing Deprivation Indices 1991, 2001 and 2011 measure the quality of housing across statistical sectors in Belgium. The indicators are time- and space-specific and represent the proportion of population who is deprived within a statistical sector.

## 1991 Housing deprivation index

Type of residence status: Proportion of individuals who rent their dwellings.

Surface of the living quarters: Proportion of individuals living in the property smaller than 35m^2^.

Main used energy: Proportion of individuals living in dwellings without central heating.

Number of toilets: Proportion of individuals living in dwellings without toilet.

Number of bathrooms: Proportion of individuals living in dwellings without bathroom.

Number of kitchens: Proportion of individuals living in dwellings without kitchen.

Connection to telephone: Proportion of individuals living in dwellings without a landline.

## 2001 Housing deprivation index

Type of residence status: Proportion of individuals who rent their dwellings.

Surface of the living quarters: Proportion of individuals living in the property smaller than 35m^2^.

Main used energy: Proportion of individuals living in dwellings without central heating.

Double glazing insulation: Proportion of individuals living in dwellings without a double-glazed insulation.

Number of toilets: Proportion of individuals living in dwellings without toilet.

Number of bathrooms: Proportion of individuals living in dwellings without bathroom.

Number of kitchens: Proportion of individuals living in dwellings without kitchen.

Connection to internet: Proportion of individuals living in dwellings without an internet connection.

## 2011 Housing deprivation index

Type of residence status: Proportion of individuals who rent their dwellings.

Main used energy: Proportion of individuals living in dwellings without central heating.

Number of bathrooms: Proportion of individuals living in dwellings without bathroom.

Number of rooms per person: Proportion of individuals living in dwellings with less than 0.5 rooms per person.

## Factor Analysis

Maximum likelihood (ML) factor analysis was applied to all indicators in the 1991, 2001, and 2011 HDI. ML factor analysis is suitable when the indicators are measured on different scales with different level of accuracy, or have different distributions. It also takes into account the ‘double-counting’ issue within the housing deprivation indices. For instance, an individual living in a dwelling without toilet is more likely to live in a dwelling without bathroom as well. Factor analysis was used to generate weights for indicators (Table S1) that were further combined with indicators to form housing deprivation scores. They were then ranked and assigned to deciles in a way that the most deprived statistical sectors fall into the first decile.

**Table S1 Indicator weights generated by factor analysis for the individual years.**

|  | Indicator weights | | |  |
| --- | --- | --- | --- | --- |
|  | **1991** | **2001** | **2011** | |
| Tenants | 3% | 11% | 3% | |
| Property size less than 35m2 | 4% | 19% |  | |
| No central heating | 15% | 18% | 43% | |
| No toilet | 17% | 16% |  | |
| No bathroom | 65% | 23% | 51% | |
| No landline | 7% |  |  | |
| No insulation |  | 23% |  | |
| No internet |  | 21% |  | |
| No kitchen | 3% | 7% |  | |
| Less than 0.5 rooms per person |  |  | 6% | |

## Missing data

We imputed missing values based on the same housing profile – individuals in the same statistical sector had to share at least 75% of housing characteristics.

**Table S2 Missing values across indicators prior and after imputing based on similar profile**

|  | 1991 | | 2001 | | 2011 | |
| --- | --- | --- | --- | --- | --- | --- |
|  | % of missing values prior imputation | % of missing values after imputation | % of missing values prior imputation | % of missing values after imputation | % of missing values prior imputation | % of missing values after imputation |
| Tenants | 5.41 | 3.8 | 7.04 | 4.0 | 2.2 | 1.03 |
| Property size less than 35m2 | 4.21 | 3.6 | 13.0 | 7.8 |  |  |
| No central heating | 6.1 | 4.2 | 7.39 | 4.7 | 12.4 | 5.8 |
| No toilet | 4.28 | 3.6 | 7.25 | 5.4 |  |  |
| No bathroom | 4.82 | 3.8 | 6.43 | 4.7 | 7.18 | 4.2 |
| No landline | 12.0 | 5.2 |  |  |  |  |
| No insulation |  |  | 8.67 | 5.7 |  |  |
| No internet |  |  | 16.7 | 9.4 |  |  |
| No kitchen | 4.28 | 2.0 | 12.8 | 7.9 |  |  |
| Less than 0.5 rooms per person |  |  |  |  | 5.5 | 2.7 |

# Mortality attributable to housing inequality

We have also calculated the percentage of mortality attributable to housing inequality by the HDI 1991, 2001, and 2011, by sex and age group in Belgium in 1991-2000, 2001-2010, 2011-2020.


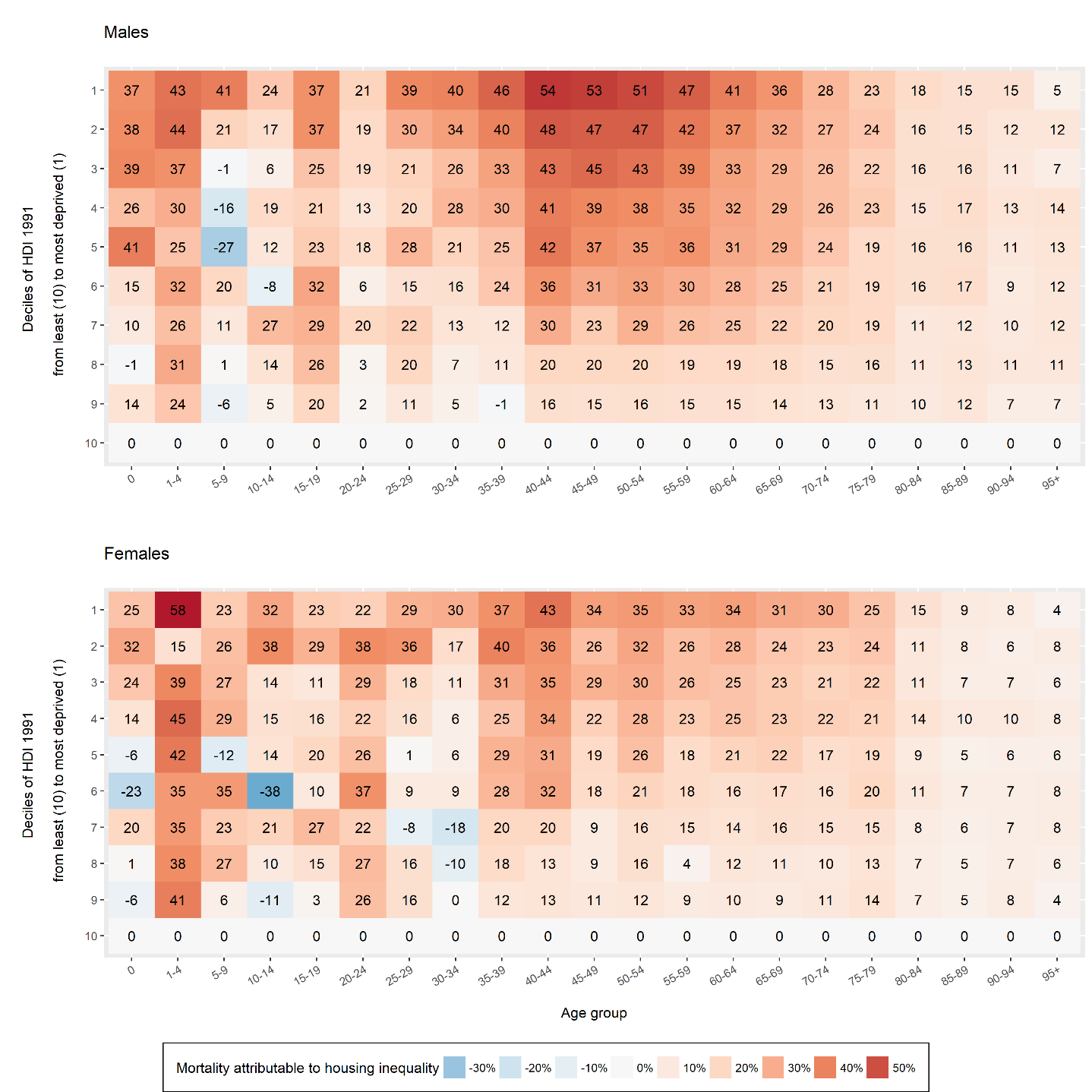


**Figure S1 Percentage of mortality attributable to housing inequality by the HDI 1991, sex and age group in a period of 1991-2000**


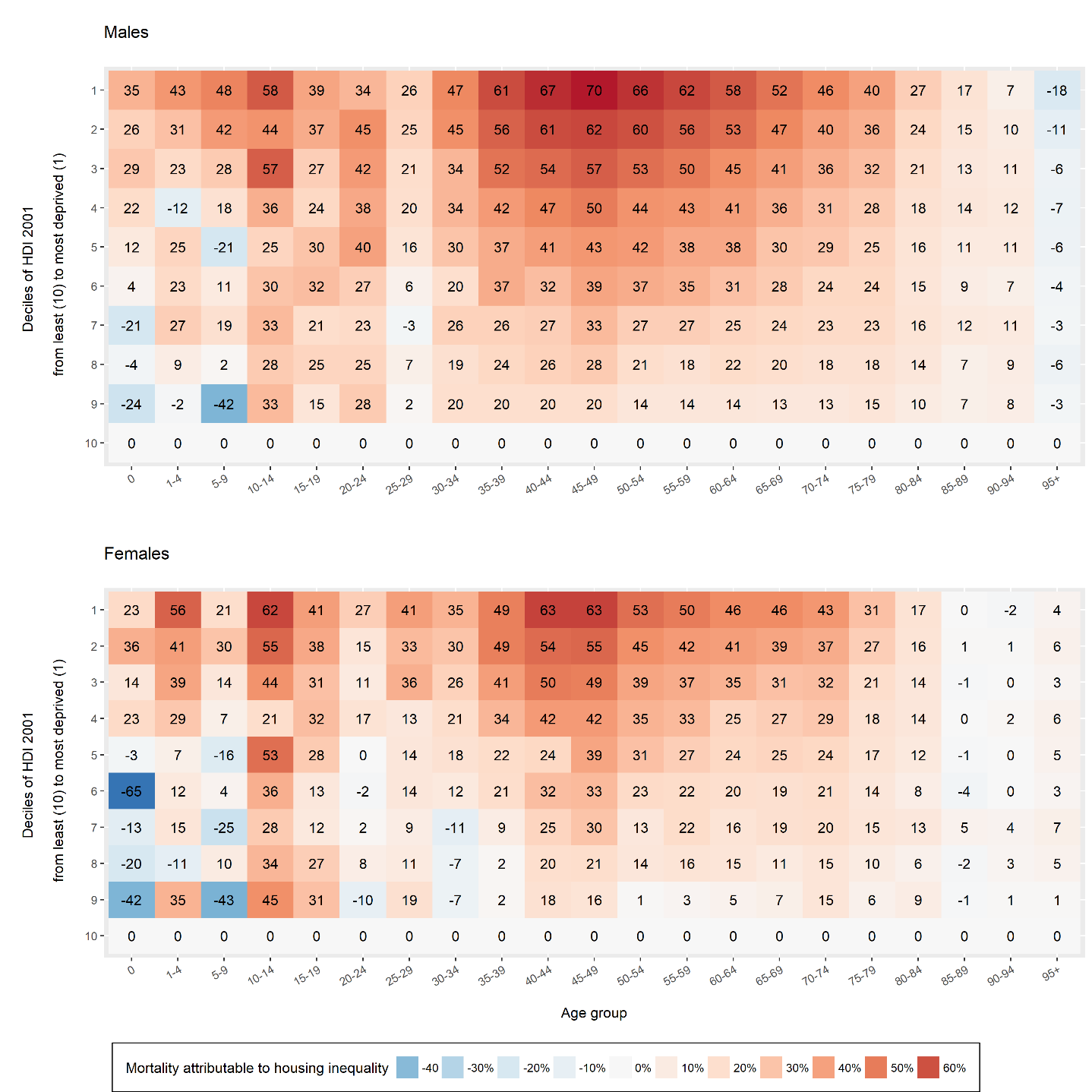


**Figure S2 Percentage of mortality attributable to housing inequality by the HDI 2001, sex and age group in a period of 2001-2010**


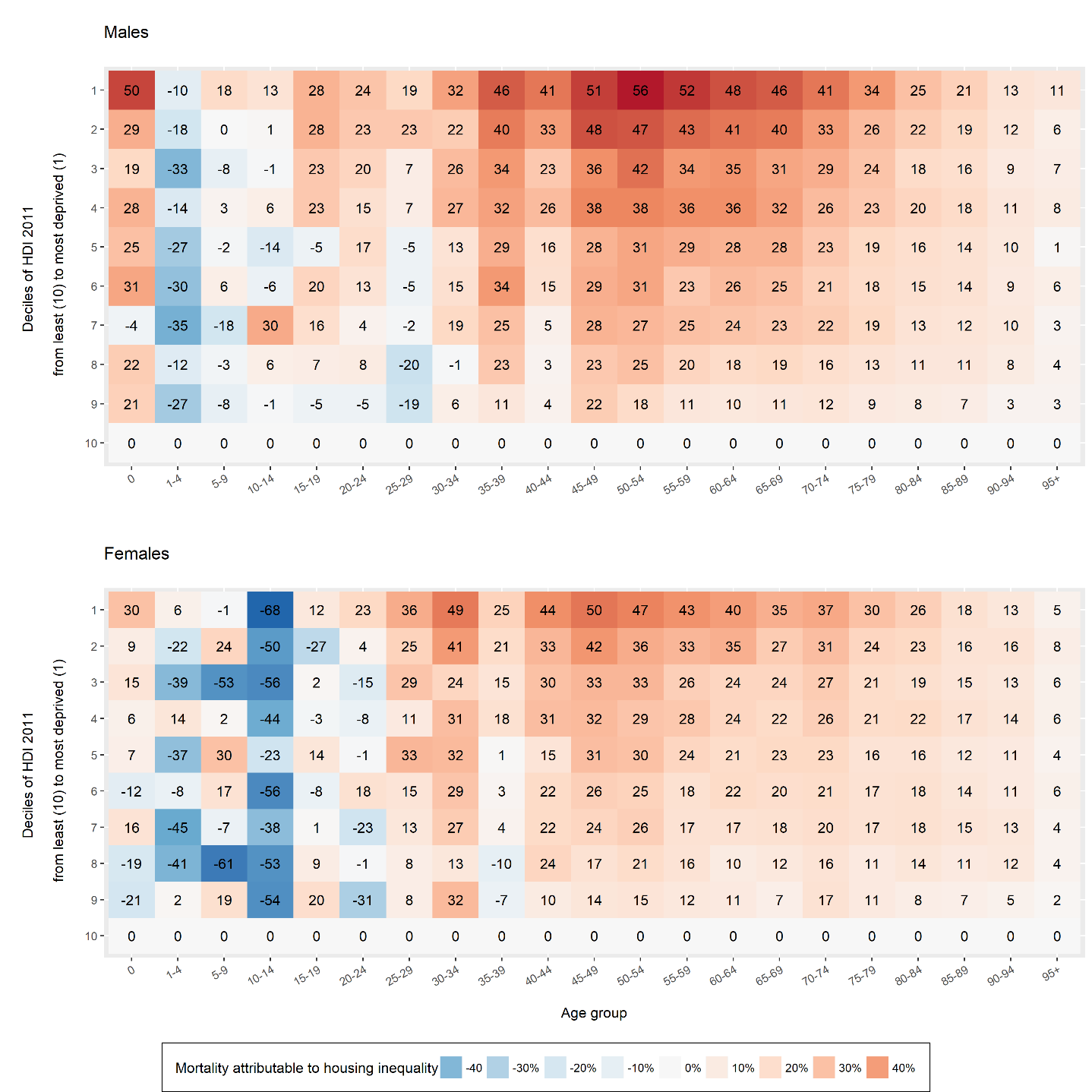


**Figure S3 Percentage of mortality attributable to housing inequality by the HDI 2011, sex and age group in a period of 2011-2020**

# Arriaga Decomposition

We constructed standard life tables for each sex, housing deprivation decile and a 10-year period using mortality rates by 5-year age group with the open-ended age interval 95+. We followed life table construction described elsewhere [1]. When converting a death rate into a probability of dying we calculated vales of _n_a_x_ using Coale and Demeny equations under age 5, and borrowed from Keyfitz and Flieger above age 5. Consequently, using Arriaga method, we compared how differences in age-specific mortality contributed to the life expectancy gap between the most and the least deprived deciles in each period of the study (Table S3).

**Table S3 Results of the Arriaga method. Absolute contribution represents the sum of the direct and indirect effects of an age group between the most and the least deprived deciles in each period.**

|  | **1991-2000** | | **2001-2010** | | **2011-2020** | |
| --- | --- | --- | --- | --- | --- | --- |
| **Age group** | **Absolute**  **contribution** | **Relative**  **Contribution**  **(%)** | **Absolute**  **contribution** | **Relative**  **Contribution**  **(%)** | **Absolute**  **contribution** | **Relative**  **Contribution**  **(%)** |
| **Males** |  |  |  |  |  |  |
| 0 | 0.07 | 1.28 | 0.03 | 0.47 | 0.04 | 0.74 |
| 0-4 | 0.08 | 1.46 | 0.05 | 0.72 | 0.00 | 0.00 |
| 5-9 | 0.04 | 0.79 | 0.03 | 0.43 | 0.01 | 0.15 |
| 10-14 | 0.02 | 0.30 | 0.05 | 0.65 | 0.01 | 0.11 |
| 15-19 | 0.09 | 1.75 | 0.08 | 1.19 | 0.04 | 0.73 |
| 20-24 | 0.11 | 2.01 | 0.09 | 1.33 | 0.05 | 0.95 |
| 25-29 | 0.21 | 4.02 | 0.07 | 1.01 | 0.04 | 0.82 |
| 30-34 | 0.20 | 3.78 | 0.15 | 2.08 | 0.08 | 1.51 |
| 35-39 | 0.23 | 4.35 | 0.26 | 3.63 | 0.14 | 2.79 |
| 40-44 | 0.30 | 5.57 | 0.43 | 6.11 | 0.18 | 3.54 |
| 45-49 | 0.35 | 6.47 | 0.67 | 9.48 | 0.29 | 5.80 |
| 50-54 | 0.39 | 7.34 | 0.80 | 11.36 | 0.48 | 9.65 |
| 55-59 | 0.49 | 9.20 | 0.88 | 12.48 | 0.60 | 12.12 |
| 60-64 | 0.57 | 10.60 | 0.92 | 13.04 | 0.67 | 13.45 |
| 65-69 | 0.58 | 10.85 | 0.86 | 12.11 | 0.71 | 14.19 |
| 70-74 | 0.47 | 8.72 | 0.73 | 10.31 | 0.65 | 13.12 |
| 75-79 | 0.46 | 8.54 | 0.57 | 8.06 | 0.49 | 9.89 |
| 80-84 | 0.40 | 7.54 | 0.28 | 3.92 | 0.31 | 6.17 |
| 85-89 | 0.22 | 4.08 | 0.10 | 1.44 | 0.17 | 3.47 |
| 90-94 | 0.07 | 1.27 | 0.02 | 0.22 | 0.04 | 0.88 |
| 95+ | 0.00 | 0.09 | 0.00 | 0 | 0.00 | 0.06 |
| **Total gain** | **5.35** |  | **7.07** |  | **4.98** |  |
|  |  |  |  |  |  |  |
|  | **1991-2000** | | **2001-2010** | | **2011-2020** | |
| **Age group** | **Absolute**  **contribution** | **Relative**  **Contribution**  **(%)** | **Absolute**  **contribution** | **Relative**  **Contribution**  **(%)** | **Absolute**  **contribution** | **Relative**  **Contribution**  **(%)** |
| **Female** |  |  |  |  |  |  |
| 0 | 0.03 | 0.88 | 0.02 | 0.40 | 0.02 | 0.48 |
| 0-4 | 0.07 | 2.53 | 0.06 | 1.59 | 0.00 | 0.12 |
| 5-9 | 0.01 | 0.42 | 0.01 | 0.23 | 0.00 | 0.00 |
| 10-14 | 0.02 | 0.84 | 0.04 | 0.96 | 0.00 | 0.00 |
| 15-19 | 0.03 | 0.98 | 0.04 | 1.04 | 0.01 | 0.24 |
| 20-24 | 0.02 | 0.85 | 0.03 | 0.73 | 0.02 | 0.60 |
| 25-29 | 0.04 | 1.41 | 0.06 | 1.39 | 0.03 | 1.00 |
| 30-34 | 0.06 | 2.12 | 0.05 | 1.36 | 0.07 | 2.01 |
| 35-39 | 0.09 | 3.25 | 0.12 | 3.03 | 0.04 | 1.33 |
| 40-44 | 0.18 | 6.15 | 0.26 | 6.61 | 0.12 | 3.60 |
| 45-49 | 0.17 | 5.88 | 0.37 | 9.27 | 0.22 | 6.54 |
| 50-54 | 0.21 | 7.20 | 0.39 | 9.93 | 0.27 | 8.22 |
| 55-59 | 0.25 | 8.67 | 0.41 | 10.31 | 0.33 | 9.88 |
| 60-64 | 0.31 | 10.65 | 0.44 | 11.11 | 0.37 | 11.19 |
| 65-69 | 0.34 | 11.61 | 0.52 | 13.07 | 0.35 | 10.53 |
| 70-74 | 0.38 | 13.15 | 0.54 | 13.62 | 0.44 | 13.22 |
| 75-79 | 0.35 | 12.13 | 0.41 | 10.31 | 0.38 | 11.62 |
| 80-84 | 0.21 | 7.17 | 0.21 | 5.29 | 0.36 | 10.97 |
| 85-89 | 0.08 | 2.83 | 0.00 | 0.00 | 0.21 | 6.42 |
| 90-94 | 0.04 | 1.23 | 0.00 | 0.00 | 0.08 | 2.47 |
| 95+ | 0.00 | 0.05 | 0.00 | 0.05 | 0.00 | 0.13 |
| **Total gain** | **2.91** |  | **3.97** |  | **3.31** |  |

# Lorenz Curve and Gini Index

Table S4 shows an example of our calculations used for plotting the adjusted Lorenz curve and Figure S4 demonstrates the outcome – the adjusted Lorenz curve for age-standardized male mortality using housing deprivation deciles (1991-2001). The points for x-axis correspond to the cumulative proportion of male population in each decile, whereas the points for y-axis correspond to cumulative proportion of adjusted number of deaths. The method of calculation was described by Martens et al. [2]

**Table S4 Example of our calculations used for plotting the adjusted Lorenz curve.**

| \| **Housing deprivation deciles** \| **ASMR** \| **Population** \| **Cumulative proportion of  population** \| **Adjusted number of deaths** \| **Cumulative  proportion of adjusted number of deaths** \| \| --- \| --- \| --- \| --- \| --- \| --- \| \| **1 (most deprived)** \| 1649.15 \| 484311.5 \| 0.09 \| 7987.04 \| 0.10 \| \| **2** \| 1598.57 \| 559081 \| 0.19 \| 8937.33 \| 0.21 \| \| **3** \| 1562.00 \| 578600.5 \| 0.30 \| 9037.73 \| 0.32 \| \| **4** \| 1549.24 \| 570876 \| 0.40 \| 8844.22 \| 0.43 \| \| **5** \| 1525.81 \| 596396.5 \| 0.51 \| 9099.90 \| 0.55 \| \| **6** \| 1493.63 \| 539086 \| 0.61 \| 8051.92 \| 0.65 \| \| **7** \| 1441.08 \| 552733.5 \| 0.72 \| 7965.31 \| 0.75 \| \| **8** \| 1395.61 \| 552782 \| 0.82 \| 7714.70 \| 0.84 \| \| **9** \| 1351.02 \| 570609 \| 0.92 \| 7709.03 \| 0.94 \| \| **10 (least deprived)** \| 1193.40 \| 417689 \| 1.00 \| 4984.70 \| 1.00 \| \|  \|  \| 5422165 \|  \| 80331.89 \|  \| |  |  |  |  |  |  |  |  |
| --- | --- | --- | --- | --- | --- | --- | --- | --- | --- | --- | --- | --- | --- | --- | --- | --- | --- | --- | --- | --- | --- | --- | --- | --- | --- | --- | --- | --- | --- | --- | --- | --- | --- | --- | --- | --- | --- | --- | --- | --- | --- | --- | --- | --- | --- | --- | --- | --- | --- | --- | --- | --- | --- | --- | --- | --- | --- | --- | --- | --- | --- | --- | --- | --- | --- | --- | --- | --- | --- | --- | --- | --- | --- | --- | --- | --- | --- | --- | --- | --- |
|  |  |  |  |  |  |  |  |  |
|  |  |  |  |  |  |  |  |  |
| 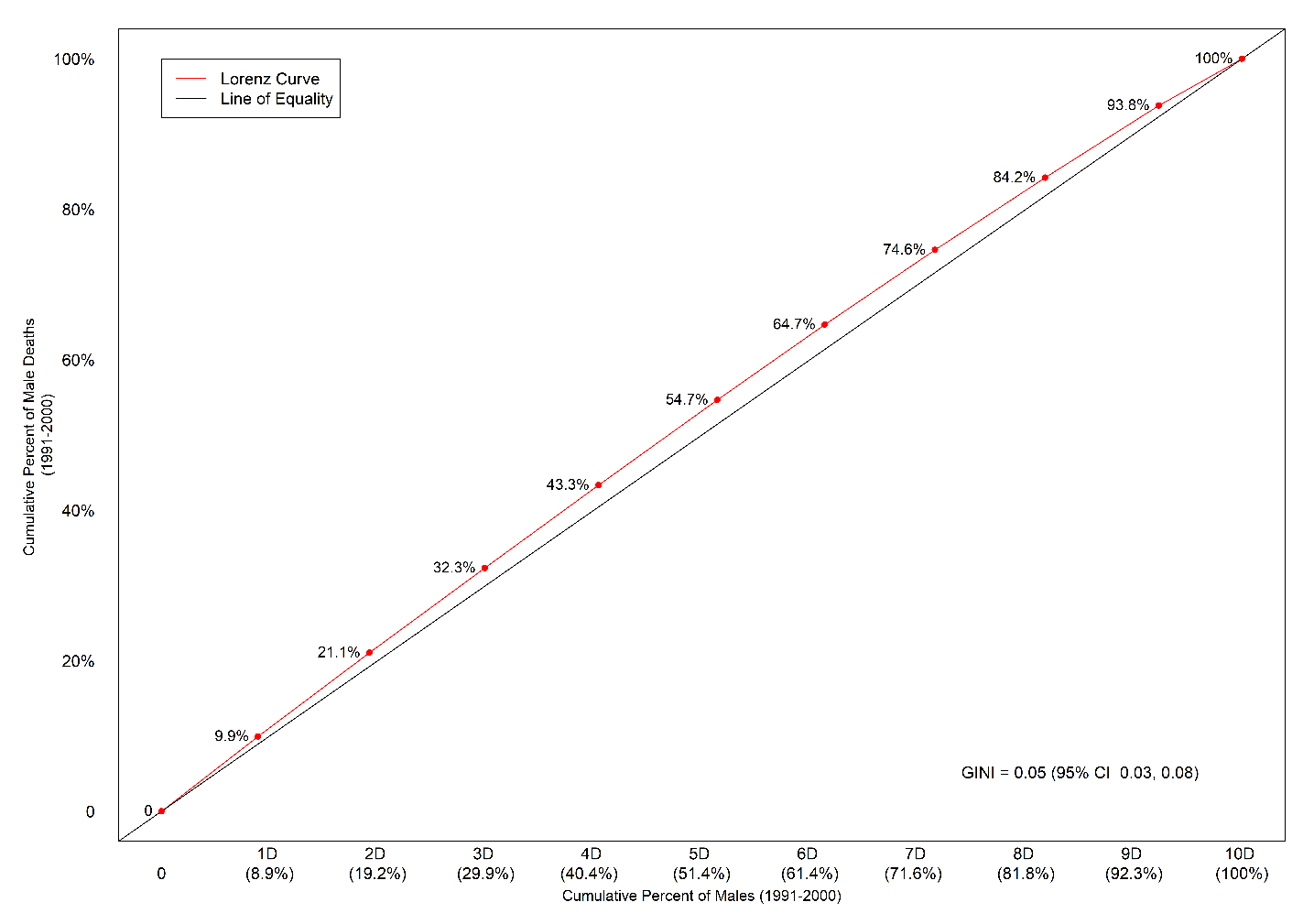 |  |  |  |  |  |  |  |  |
| **Figure S4 Adjusted Lorenz curve for age-adjusted male mortality in the period between 1991-2000** |  |  |  |  |  |  |  |  |


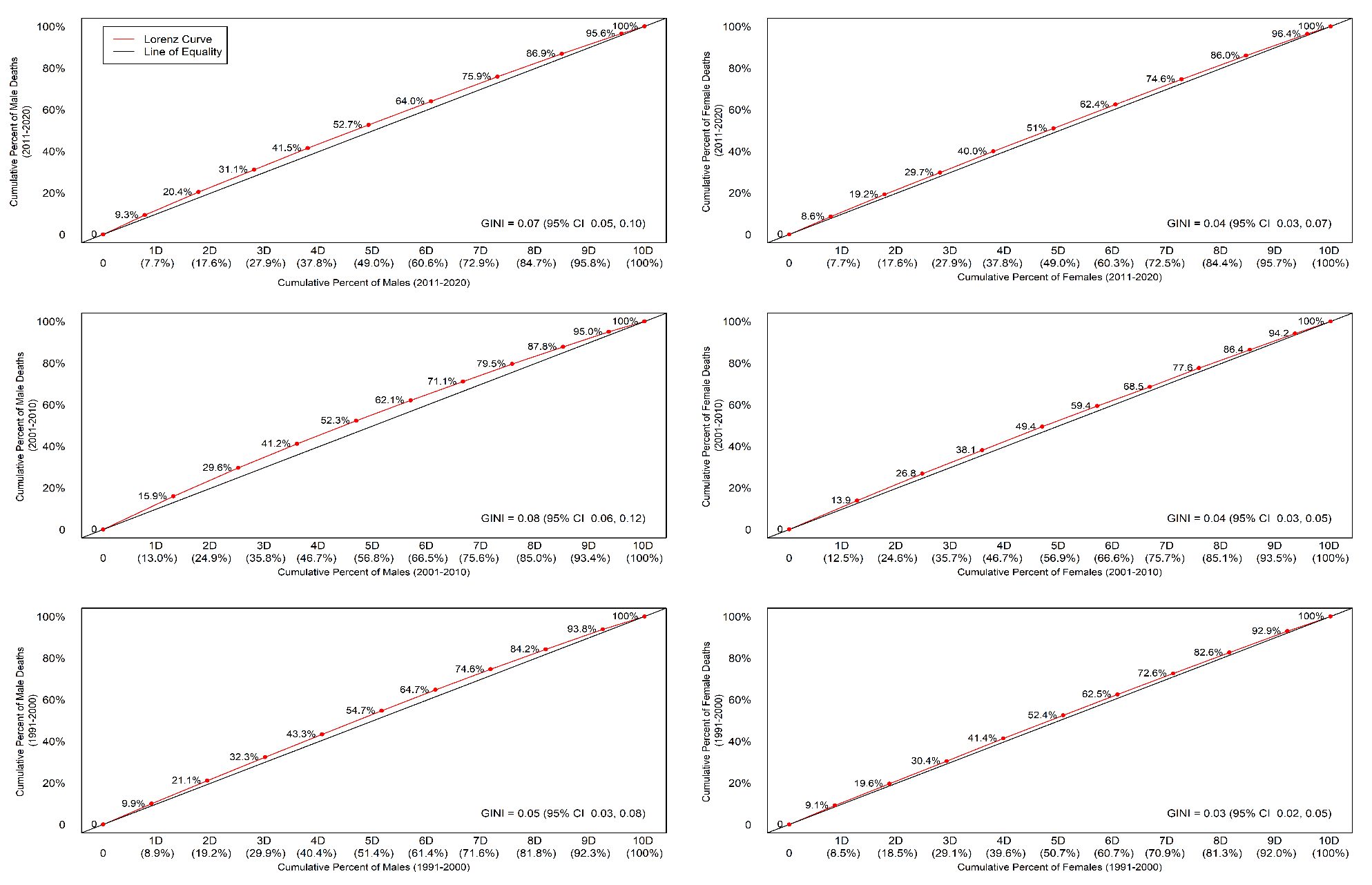


**Figure S5** **Adjusted Lorenz curves for male and female mortality in the period between 1991-2000, 2001-2010, and 2011-2020**

# Spearman’s Rank Correlation

**Table S5 Spearman’s correlation coefficients between the indicators of the HDI 1991 and HDI 2001.**

|  | **Tenants** | **Property size < 35m2** | **No central heating** | **No toilet** | **No bathroom** | **No kitchen** |
| --- | --- | --- | --- | --- | --- | --- |
| **Tenants** | 0.81 |  |  |  |  |  |
| **Property size < 35m2** |  | 0.41 |  |  |  |  |
| **No central heating** |  |  | 0.84 |  |  |  |
| **No toilet** |  |  |  | 0.42 |  |  |
| **No bathroom** |  |  |  |  | 0.65 |  |
| **No kitchen** |  |  |  |  |  | 0.96 |

**Table S6 Spearman’s correlation coefficients between the indicators of the HDI 2001 and HDI 2011.**

|  | **Tenants** | **No central heating** | **No bathroom** |
| --- | --- | --- | --- |
| **Tenants** | 0.76 |  |  |
| **No central heating** |  | 0.79 |  |
| **No bathroom** |  |  | 0.65 |

1. Preston, S.H., P. Heuveline, and M. Guillot, *Demography - Meausing and Modeling Population Processes*. 2001: Blackwell Publishing.

2. P, M., et al., *Health Inequities in Manitoba: Is the Socioeconomic Gap in Health Widening or Narrowing Over Time?* 2010, Manitoba Centre for Health Policy: Winnipeg.
